# Listening evaluation of cochlear implant users: comparison of subjective and objective evaluation by visual analogue scale

**DOI:** 10.1017/S0022215123001445

**Published:** 2024-03

**Authors:** T Yano, R Tomioka, K Shirai, N Nishiyama, K Tsukahara

**Affiliations:** Department of Otorhinolaryngology, Head and Neck Surgery, Tokyo Medical University, Tokyo, Japan

**Keywords:** Cochlear implant, visual analogue scale

## Abstract

**Objective:**

This study aimed to use short-form visual analogue scale cochlear implantation questionnaires to evaluate subjective aspects at each out-patient visit. The correlation between subjective hearing tests using the short-form visual analogue scale and objective hearing outcomes was evaluated.

**Method:**

This study was conducted in a single centre. Cochlear implant users (*n* = 199) evaluated their hearing on a scale of 0 to 100 for the right, left and both ears. The Japanese speech perception test (CI-2004) Japanese monosyllable speech perception test (67-S) and cochlear implantation threshold were used for the objective cochlear implantation evaluation.

**Results:**

A significant correlation was found between the short-form visual analogue scale questionnaire and objective hearing outcome, for words (*r* = 0.64) and sentences (*r* = 0.62) in CI-2004 and 67-S (*r* = 0.56) tests. No significant correlation was found between the short-form visual analogue scale score and cochlear implantation threshold (*r* = −0.18).

**Conclusion:**

Short-form visual analogue scale cochlear implantation questionnaires mean cochlear implant users spend less time answering subjective visual analogue scale questionnaires, and clinicians estimate a patient's cochlear implantation hearing and abnormality by chronological evaluation.

## Introduction

Our department performed its first cochlear implantation in Japan in 1985. Since then, we have consistently provided cochlear implantation and pre-operative and post-operative hearing, instrumentation and language training. There have been many previous studies^1–6^ on the effectiveness of cochlear implantation, and various items have been evaluated. Improved hearing is one of the most important goals of cochlear implantation, and today, word audibility using the CI-2004 test is widely used as a simple, easily comparable and objective method for evaluating the effectiveness of cochlear implantation in daily practice. However, laboratory testing does not reflect daily life and the importance of assessing functional outcomes, that is, ‘how cochlear implants function in daily life and how the wearer communicates’, has been emphasised.^7–9^ Therefore, our department has emphasised the evaluation of functional outcomes of cochlear implant wearers and has conducted ongoing surveys to examine subjective evaluations at various ages.^10^

Although detailed questionnaires provide information on the daily life of the wearer, they are time-consuming, difficult to conduct frequently and unsuitable for capturing detailed day-to-day changes. In addition, examinations of cochlear implant users are time-consuming because of the many tests that must be performed, including telemetry tests, program adjustments and audibility tests. The hearing of cochlear implant users changes in the early post-operative period and stabilises within a year,^11^ but it continues to change for various reasons, such as equipment failure. Therefore, to improve the efficiency of out-patient care, we asked patients to perform a brief self-assessment of their listening comprehension using the visual analogue scale (VAS) when waiting for each examination; this was then used for examination planning and hearing management to make effective use of the limited examination time.

In this study, the effectiveness of VAS in hearing management was examined by comparing self-assessment by VAS with objective assessment in a conventional laboratory setting with regard to hearing performance in cochlear implant cases.

## Materials and methods

Of the 1120 cases who underwent cochlear implantation at the Tokyo Medical University Hospital between November 1985 and March 2021, those who were at least 20 years old as of March 2021 were included. Hearing and listening self-assessments were conducted on eligible patients on the same day, as described below. A total of 199 patients met the study criteria and were enrolled. Exclusion criteria were lack of either subjective or objective assessments.

**Figure 1.**
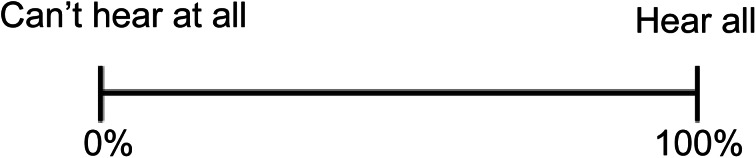
Questionnaire used in our hospital.

As a subjective evaluation, the patient was asked to rate his or her listening for words in the right ear, the left ear and both ears using the VAS (Figure 1). At the same time, the clinician asked about any changes or difficulties experienced since the last visit.

Objective listening assessments included the cochlear implant threshold, the monosyllable speech perception 67-S test under cochlear implantation, and the adult word and adult sentences sections of the Japanese speech perception test CI-2004. In binaural cases, the results for the right ear, left ear and both ears were used.

The relationship between the subjective and objective ratings was analysed using Pearson's correlation co-efficient. For the statistical analysis, SPSS® (version 21.0) statistical analysis software was used. This study was approved by the Medical Research Ethics Review Committee of Tokyo Medical University (reception number: 3725).

## Results

### Patient background

The respondents ranged in age from 20–87 years, with a mean age of 58.5 years. There were 110 cases involving the right ear, 50 involving the left ear and 39 involving both ears.

### Self-evaluation by visual analogue scale

The VAS rated verbal hearing for the right ear as ranging from 5 to 100 per cent, with a mean of 72.2 per cent, for the left ear as ranging from 3 to 96 per cent, with a mean of 63.3 per cent, and for binaural cases as ranging from 20 to 100 per cent, with a mean of 76.8 per cent.

### Objective evaluation

Wearing thresholds for one ear were 14–61 dB (mean, 28.5 dB) for the right ear, 19–50 dB (mean, 31.5 dB) for the left ear and 20–56 dB (mean, 28.3 dB) for both ears.

The best speech intelligibility under cochlear implantation, as assessed by the 67-S test ranged from 0–96 per cent with the right implant (mean, 64.1 per cent), 5–90 per cent with the left implant (mean, 58.6 per cent) and 15–100 per cent with binaural implants (mean, 71.1 per cent).

Word comprehension, as assessed by the CI-2004 adult words test ranged from 16–100 per cent with the right ear (mean, 73.7 per cent), 0–100 per cent with the left ear (mean, 65.9 per cent) and 20–100 per cent in binaural cases (mean, 82.3 per cent).

Sentence comprehension, as assessed by the CI-2004 adult sentences test ranged from 20–100 per cent with right cochlear implantation (mean, 78 per cent), 0–100 per cent with left cochlear implantation (mean, 71.5 per cent) and 20–100 per cent with binaural implant wearers (mean, 84.6 per cent).

### Relation between self-assessment and objective assessment

**Figure 2.**
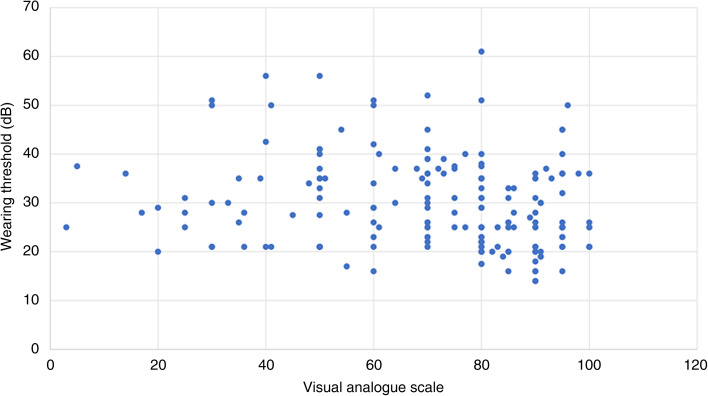
Visual analogue scale and wearing threshold.

**Figure 3.**
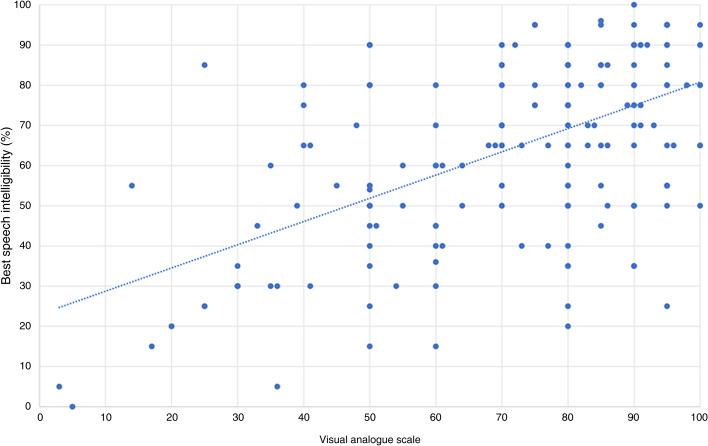
Visual analogue scale and best speech intelligibility.

**Figure 4.**
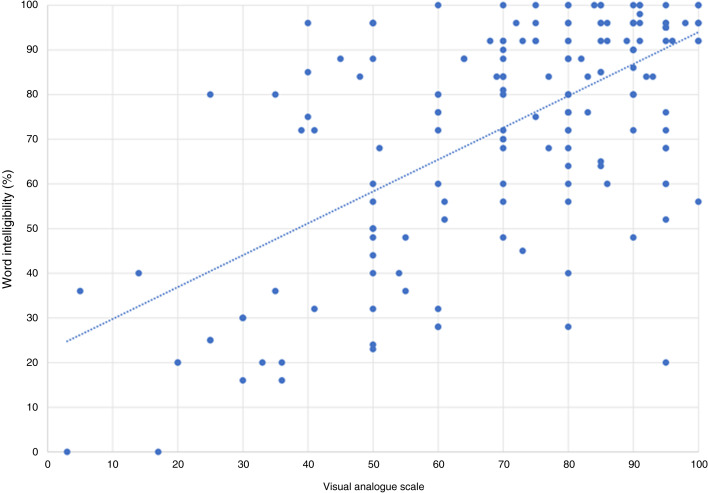
Visual analogue scale and word intelligibility.

**Figure 5.**
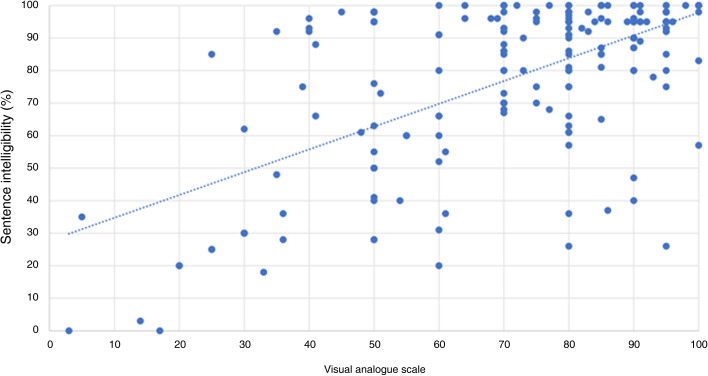
Visual analogue scale and sentence intelligibility.

There was no correlation between the VAS and cochlear implant threshold for speech perception, with a correlation co-efficient of −0.176 (*p* = 0.013). There was a positive correlation between the VAS and best speech intelligibility with a correlation co-efficient of 0.565 (*p* < 0.001); between the VAS and word comprehension with a correlation co-efficient of 0.634 (*p* < 0.001): and between the VAS and sentence comprehension with a correlation co-efficient of 0.431 (*p* < 0.001) (Figures 2–5).

## Discussion

The highest speech intelligibility, word comprehension and sentence comprehension under cochlear implantation were significantly related to the self-assessment of speech listening (VAS). The first advantage of this questionnaire is that it is very simple to answer, has no complex questions and can be administered during an out-patient waiting period. The questionnaire can be administered at or before the time of the examination, facilitating the planning of the day's examination and rehabilitation and can be useful for detecting abnormalities and for efficient examination by evaluating the patient over time during each out-patient visit.

Although the objective assessment that correlated with the present study is a routine test, provides easily collectable data and is excellent for comparison with other tests, it must be understood that this questionnaire does not necessarily represent functional outcomes because it does not reflect the actual life of the patient. The Nijmegen Cochlear Implant Questionnaire and the Speech, Spatial, and Qualities of Hearing Scale^12^ have been developed and reported as self-assessment questionnaires for cochlear implant patients for subjective evaluation of cochlear implants. Although these questionnaires are time-consuming and include detailed self-assessment questions, they have the advantage of providing a measure of functional outcomes and an assessment of how well the cochlear implant wearer uses the implant in daily life and how well it helps them communicate. In addition, studies using questionnaires to check post-operative quality of life^13,14^ have been conducted, and attempts have been made to understand the status of patients using questionnaires. In contrast, the VAS evaluation conducted at our clinic takes only a few minutes for self-assessment and correlates to some extent with objective assessment, which has the advantage of allowing us to detect and respond to hearing changes in patients with cochlear implants to some extent, without having to perform multiple objective assessments each time. However, unlike the aforementioned questionnaires, it is difficult to express the functional outcomes. Therefore, the VAS questionnaire should be used as a simple indicator of changes in cochlear function, while the functional outcome questionnaire should be administered once every six months or once a year to obtain a more detailed understanding of the status of daily use. The questionnaire was used only for overall evaluation. The questionnaire only assessed the overall status of the patients, but a more detailed classification, such as the cause of hearing loss, the duration of cochlear implant use and the duration of hearing loss could lead to differences in the assessment of functional outcomes. In addition, since the objective test was conducted under quiet conditions, there is room for further study on the correlation between the results of the objective test and those of the test conducted under noisy conditions that are more common in daily use.

Although the present study was able to demonstrate the relationship between self-evaluation (VAS) and objective evaluation, there were some cases in which self-evaluation and objective evaluation diverged. In such cases, it is necessary to confirm the factors that cause the self-evaluation to decrease in each case.

Although subjective evaluation can provide a sense of how a person feels in his or her own life, it is necessary to consider the possibility of large sensory differences among individuals, because psychogenic factors are involved. Therefore, it is necessary to examine the changes over time.

## Conclusion

The relationship between a brief self-assessment using a questionnaire and a conventional listening assessment of the implant's listening performance was examined. This study found that the 67-S, CI-2004 word test and CI-2004 sentence test and self-assessment listening were related. A simple self-assessment when the patient is waiting for examination showed the possibility of understanding current listening status, which may be useful for detecting abnormalities and for efficient examination.

- This study evaluated subjective functional outcomes in several self-reported questionnaires
- These questionnaires have several detailed sections, and it takes time to answer all the questions
- This study set up short-form visual analogue scale (VAS) cochlear implant (CI) questionnaires
- The correlation between subjective hearing tests using the short-form VAS and objective hearing outcomes was evaluated
- This study found a significant correlation between the short-form VAS scale questionnaire and objective hearing outcome
- Clinicians can estimate a patient's hearing with cochlear implants and abnormality on every out-patient occasion using chronological evaluation
